# Respiratory syncytial virus-induced ADAM17 activity controls maintenance of epithelial barrier integrity and promotes syncytium formation

**DOI:** 10.1128/jvi.01150-26

**Published:** 2026-09-28

**Authors:** Robert Meineke, Marie-Christin Knittler, Rajarshee Tagore, Nele Engling, Martin Ludlow, Albert D. M. E. Osterhaus, Guus F. Rimmelzwaan

**Affiliations:** 1 Research Center for Emerging Infections and Zoonoses, University of Veterinary Medicine Hannover 686574, Hanover, Germany; University of Minnesota Twin Cities, Minneapolis, Minnesota, USA

**Keywords:** RSV, ADAM17, epithelial barrier, syncytial formation, EGFR

## Abstract

Respiratory syncytial virus (RSV) infection causes epithelial injury, barrier disruption, and formation of syncytia in human lung tissue; however, the regulatory host defense mechanisms involved remain poorly defined. ADAM17 is a multifunctional sheddase implicated in inflammatory lung disease and is considered a potential therapeutic target during viral infections. Here, we show that RSV infection induces ADAM17 activity through PKC- and ERK-dependent signaling, increasing surface ADAM17 levels and sheddase activity. Genetic loss or pharmacological inhibition of ADAM17 reduced RSV-induced formation of syncytia, post-entry viral spread, and viral titers. This identifies ADAM17 as a host factor that supports viral dissemination. ADAM17 deficiency simultaneously exacerbated RSV-induced epithelial injury, characterized by loss of intercellular cohesion, impaired barrier function, increased paracellular permeability, and enhanced cytotoxicity. RSV-induced ADAM17 activity promoted the release of multiple epithelial growth factor receptor (EGFR) ligands. Supplementation with recombinant amphiregulin partially restored barrier function in infected ADAM17-deficient cells. Consistent with these functional effects, ADAM17 deficiency was associated with the loss of epithelial differentiation-associated transcriptional programs and increased expression of inflammatory genes. These findings indicate that ADAM17 plays a dual role in RSV infection. It facilitates syncytial viral spread, while preserving epithelial integrity through autocrine EGFR signaling. Thus, ADAM17 activity represents a biological trade-off between viral dissemination and tissue protection, indicating that therapeutic ADAM17 inhibition may reduce viral spread at the cost of aggravating epithelial damage.

**IMPORTANCE:**

During respiratory syncytial virus (RSV) infection, the airway epithelium must maintain barrier integrity while responding to virus-induced syncytium formation and tissue damage. It is poorly understood which host factors balance tissue preservation against viral replication and spread. Here, we demonstrate that the host cellular metalloproteinase ADAM17 is activated upon RSV infection. While RSV-induced ADAM17 activity promotes syncytium formation and viral spread, it concurrently controls the maintenance of epithelial barrier integrity, preserves cell survival, and inhibits hyperinflammatory signaling. By demonstrating that ADAM17 has a dual mode of action controlling both viral propagation and epithelial barrier resilience, this work uncovers a central trade-off: the same host factor exploited by RSV for dissemination is essential for protecting airway tissue. Consequently, targeting ADAM17 for antiviral therapy presents major challenges, as blocking viral spread may compromise vital epithelial defenses.

## INTRODUCTION

Respiratory syncytial virus (RSV) is a major cause of acute lower respiratory tract infections with a substantial disease burden in young children, older adults, and immunocompromised individuals (1–3). Annually, RSV accounts for over 3.6 million hospitalizations worldwide and approximately 100,000 deaths in children under 5 years of age (1). Recently, monoclonal antibodies and vaccines have become available to prevent severe RSV infections (4–6). Therapeutic options that alleviate clinical symptoms once an infection is established remain limited, and the molecular factors that determine disease severity are incompletely understood (6).

The airway epithelium is both the primary site of RSV replication and the first line of defense against respiratory pathogens (7). RSV infects ciliated and non-ciliated airway epithelial cells and drives the formation of multinucleated syncytia through fusion (F) protein-mediated cell-to-cell fusion that mediates viral spread, which is a hallmark of RSV pathology (4, 7). Concurrently with syncytium formation, RSV infection disrupts tight junction integrity, increases paracellular permeability, compromises barrier function, and facilitates pathogen access to submucosal tissue (8). Although these processes are well documented, the molecular links between viral replication and epithelial remodeling remain poorly defined.

A disintegrin and metalloproteinase 17 (ADAM17), also known as TNF-α converting enzyme (TACE), is a transmembrane sheddase that cleaves the ectodomains of more than 80 membrane-anchored substrates, including cytokines, cytokine receptors, adhesion molecules, and growth factor ligands (9–11). Among the best-characterized substrates are the epithelial growth factor receptor (EGFR) ligands amphiregulin (AREG), epiregulin, and TGF-α (12); their shedding initiates paracrine and autocrine EGFR-ERK signaling with broad effects on epithelial cell proliferation, differentiation, and cell-to-cell junction formation (13). ADAM17 activity is regulated post-translationally by PKC and MEK/ERK signaling and is subject to spatial control at the plasma membrane, enabling rapid, context-dependent modulation of its enzymatic activity (14, 15). In the context of respiratory virus infections, ADAM17 mediates ACE2 shedding during SARS-CoV-2 infection (16, 17), and RSV infection activates EGFR signaling in airway epithelial cells with downstream effects on inflammation and apoptosis (18). Whether RSV infection induces ADAM17 activity, which upstream signals drive this induction, and how ADAM17-mediated shedding affects viral spread and epithelial barrier integrity remains incompletely understood.

The ADAM17-EGFR axis controls epithelial cell homeostasis and innate immune signaling (9–11, 19). During chronic airway disease, hyperactivation of this axis drives mucus hypersecretion, goblet cell metaplasia, and epithelial barrier remodeling (20–23). The consequences of ADAM17 activation during acute viral infection are unclear. On one hand, EGFR ligand shedding could strengthen junction integrity and promote epithelial cell survival (12, 18). On the other hand, ADAM17-mediated shedding of TNF-α and TNFRI generates a soluble decoy receptor pool that dampens local TNF-α signaling and blunts antiviral inflammatory responses (9, 24). The shedding of syndecan-1 and syndecan-4 further removes heparan sulfate proteoglycans that several respiratory pathogens, including RSV, exploit as low-affinity attachment factors, altering the availability of viral entry co-receptors (25–27). We hypothesize that RSV-induced ADAM17 activity has a dual effect of promoting viral spread while shaping the epithelial response.

In the present study, we investigated whether RSV infection induces ADAM17 activity in human airway epithelial cells and defined the upstream signaling pathway controlling it. Using an isogenic ADAM17-deficient A549 cell line, we examined the consequences of ADAM17-mediated ectodomain shedding for viral spread, epithelial barrier integrity, and the host transcriptional response to infection.

## MATERIALS AND METHODS

### ADAM17 expression analysis in the human lung

To characterize ADAM17 expression and co-expression context across human lung cell types, the Mavatar Discovery platform (v1.12.2; Mavatar AB) was used to query the Human Lung v1.0 network, which integrates publicly available transcriptomic data from lung biopsy samples and their MeSH child descriptors sourced from GEO. ADAM17 expression was visualized across annotated lung cell clusters using the UMAP-based network projection platform. Gene co-expression network analysis was performed using the DINA (Deep Integrated Network Analysis) framework with default parameters to identify genes co-expressed with ADAM17 in the human lung context. Briefly, Mavatar Discovery aggregates publicly deposited single-cell RNA-sequencing data sets from GEO into a unified UMAP embedding across annotated cell types. DINA performs correlation-based co-expression network inference on this aggregated data set to identify genes with expression patterns similar to a query gene. Gene Ontology enrichment analysis of biological processes and cellular components was conducted on the ADAM17 co-expression network, with enriched terms ranked by adjusted *P*-values.

### Cell lines and culture conditions

HEp-2 (ATCC, CCL-23) cells were cultured in minimum essential medium with Earle’s salts (Capricorn Scientific, MEM-A), supplemented with 10% fetal bovine serum (FBS, Gibco) and 1% penicillin/streptomycin (P/S, Capricorn Scientific) at 37°C and 5% CO₂. A549 (ATCC, CCL-185) cells and the isogenic ADAM17^−/−^ cell line were cultured in Ham’s F-12 medium (Capricorn Scientific), supplemented with 10% FBS (Gibco) and 1% penicillin/streptomycin (Capricorn Scientific), at 37°C and 5% CO₂. Both the A549 ADAM17^−/−^ cell line and the isogenic parental A549 wild-type (WT) cell line were purchased from Ubigene. The ADAM17-deficient line was generated by CRISPR-Cas9 genome editing using two guide RNAs targeting exon 9 of the ADAM17 locus (gRNA7: 5′-GAATCCTTGAAATACTTGAGAGG-3′; gRNA8: 5′-GCTGGGATTATTAAGATGATTGG-3′) delivered by electroporation (1,320 V, 30 ms, two pulses). Clonal selection yielded clone A4, which was confirmed by Sanger sequencing to carry a complete deletion of exon 9 across all three alleles (571 bp, 569 bp, and 577 bp deletions, respectively). The complete loss of ADAM17 protein was verified by western blot. All cell lines were STR-typed and used at passage numbers ≤20. The cells were passaged at 80–90% confluence.

### Virus propagation and stock preparation

Virus stocks were generated by infecting HEp-2 cells at 60%–80% confluency in OptiMEM (Thermo Fisher Scientific) supplemented with 100 IU/mL penicillin and 100 µg/mL streptomycin with RSV-A-0594 or rRSV-A-0594-eGFP at a multiplicity of infection (MOI) of 0.1 (28). Infected cells were incubated at 37°C and 5% CO₂ until cytopathic effects were visible (3–5 days). The virus was harvested by scraping the infected cells, followed by centrifugation at 1,000 × *g* for 10 min at 4°C to remove cell debris. The supernatants were collected and mixed with 50% sterile polyethylene glycol 6000 (PEG; Carl Roth) to a final concentration of 10% PEG. The virus-PEG mixture was incubated at 4°C for 4 h on a shaker and subsequently centrifuged at 3,000 × *g* for 30 min at 4°C. The supernatant was removed, and pellets were resuspended in Hank’s balanced salt solution (Gibco) containing 20% sucrose (Carl Roth). The virus was aliquoted, snap-frozen in liquid nitrogen, and stored at −150°C until further use. Viral titers were determined by the 50% tissue culture infectious dose (TCID_50_)/mL method on HEp-2 cells, as described by Reed and Muench (29).

### Viral replication kinetics and infectious virus titration

To assess the effect of ADAM17 on viral replication, A549 WT and A549 ADAM17^−/−^ cells were infected with RSV-A-0594 (ON1 genotype, 24-amino-acid G protein insertion) and rRSV-A-0594-eGFP (recombinant RSV-A-0594 with eGFP gene between the viral P and M genes) at MOIs of 0.1, 0.5, and 1. Supernatants were collected at 96 h post-infection (hpi), along with mock-infected controls. Infectious virus titers were determined using a standard TCID_50_/mL assay on A549 WT cells, as described above. For RSV-A-0594, plates were fixed 5 days post-infection with 4% PFA in phosphate-buffered saline (PBS) and permeabilized with 0.5% Triton X-100. Cells were blocked in PBS containing 5% BSA and incubated with polyclonal goat anti-RSV (AB1128, 1:2,500, Merck) for 1 h at room temperature, followed by HRP-conjugated donkey anti-goat IgG (A15999, 1:10,000, Invitrogen). Staining was visualized using TrueBlue peroxidase substrate (SeraCare). For rRSV-A-0594-eGFP, viral titers were determined by fluorescence microscopy using a Leica DM8 fluorescence microscope.

### RT-qPCR

Total RNA was extracted from A549 WT and A549 ADAM17^−/−^ cells using the NEB Monarch Total RNA Miniprep Kit, according to the manufacturer’s instructions. RT-qPCR was performed using NEB Luna Universal One-Step RT-qPCR Kit on a Roche LightCycler 96 system. The thermal cycling protocol consisted of four stages: reverse transcription activation at 50°C for 2 min, initial denaturation at 95°C for 10 min, 40 cycles of denaturation at 95°C for 15 s, and annealing/extension at 60°C for 1 min, followed by melting curve analysis (95°C for 15 s, 60°C for 15 s, and 95°C for 15 s). Each experiment was performed in three independent runs with two biological replicates. Gene expression was normalized to the geometric mean of three reference genes (GAPDH, PGK1, and PPIA), and relative expression was calculated using the 2^−ΔΔCt^ method. The following primer pairs used are listed in Table 1.

**TABLE 1:** qPCR primer sequences for ADAM17 compensatory target genes and normalization controls

Gene	Forward (5′ → 3′)	Reverse (5′ → 3′)
ADAM17	AAC AGC GAC TGC ACG TTG AAG G	CTG TGC AGT AGG ACA CGC CTT T
ADAM10	GAG GAG TGT ACG TGT GCC AGT T	GAC CAC TGA AGT GCC TAC TCC A
ADAM8	TGC TGG AGG TGG TGA ATC ACG T	TCA GGA GGT TCT CCA GTG TGA C
ADAM9	CTT GCT GCG AAG GAA GTA CCT G	CAC TCA CTG GTT TTT CCT CGG C
ADAM12	ATG GCA TCT GCC AGA CTC ACG A	GGA ACT CTT CGA GAC TTT GCC AC
MMP2	AGC GAG TGG ATG CCG CCT TTA A	CAT TCC AGG CAT CTG CGA TGA G
MMP9	GCC ACT ACT GTG CCT TTG AGT C	CCC TCA GAG AAT CGC CAG TAC T
MMP14	CCT TGG ACT GTC AGG AAT GAG G	TTC TCC GTG TCC ATC CAC TGG T
GAPDH	GTC TCC TCT GAC TTC AAC AGC G	ACC ACC CTG TTG CTG TAG CCA A
PGK1	CCG CTT TCA TGT GGA GGA AGA AG	CTC TGT GAG CAG TGC CAA AAG C
PPIA	GGC AAA TGC TGG ACC CAA CAC A	TGC TGG TCT TGC CAT TCC TGG A

### ADAM17 sheddase activity assay

A549 WT and A549 ADAM17^−/−^ cells were seeded at 2.5 × 10^4^ cells per well in 96-well black plates with optical bottoms (Thermo Fisher Nunc) and incubated overnight at 37°C with 5% CO₂. On day 1, the medium was replaced with serum-free FluoroBrite DMEM/F-12, and the cells were serum-starved for 4 h at 37°C and 5% CO₂. Cells were then infected with RSV-A-0594 at MOIs of 0.01, 0.1, 1, or mock-treated in FluoroBrite DMEM/F-12 for 2 h at 37°C, with gentle rocking every 30 min. After inoculum removal, the cells were incubated with marimastat (40 µM), GI254023X (2 µM), SB202190 (5 µM), U0126 (10 µM), or BIM-1 (5 µM) prepared from 10 mM stocks in FluoroBrite DMEM/F-12. The vehicle control was DMSO at the corresponding 1:250 dilution. After 30 min, the inhibitor solution was removed and replaced with fresh inhibitor-containing medium supplemented with 1 µM fluorogenic FRET peptide substrate selective for TACE/ADAM17 (TACE Substrate II, Enzo Life Sciences, cat. no. BML-P228; FAM/TAMRA fluorophore-quencher pair). Cleavage of the substrate by ADAM17 separates the FAM donor from the TAMRA quencher, generating a fluorescence signal proportional to the sheddase activity. The plates were protected from light and returned to 37°C with 5% CO₂. Fluorescence was measured at 0, 24, and 48 h after substrate addition using a Tecan Spark plate reader at Ex/Em 485/535 nm. Each experiment was performed in three independent experiments with three biological replicates each (*n* = 9 per condition).

### Live-cell imaging, proliferation kinetics, and syncytia quantification

All live-cell imaging was performed using an IncuCyte S3 live-cell imaging system (Sartorius). For proliferation kinetics, A549 WT and A549 ADAM17^−/−^ cells were seeded at 5 × 10^4^ cells per well in 24-well plates and imaged every 2 h for 72 h. Cell boundaries were detected using the label-free cell-by-cell analyzer integrated into the IncuCyte software (v10.4.1) with the following segmentation parameters: Surface Fit, Edge Split On, Hole Fill 10 µm². Cell debris was excluded by applying a minimum cell area threshold of 500 µm² and an eccentricity filter of 0.4. Proliferation rates were compared between genotypes using linear regression of cell counts over time.

For viral spread kinetics, A549 WT and A549 ADAM17^−/−^ cells were infected with rRSV-A-0594-eGFP at MOIs of 0.1, 0.5, and 1, or mock-treated, and total integrated GFP intensity was measured at 24-h intervals from 0 to 96 hpi. The area under the curve was calculated from the resulting time-course profiles to compare the cumulative viral spread between the genotypes. For syncytial quantification, cells were infected at an MOI of 1 and treated with DMSO (vehicle control; 1:250 dilution), GI254023X (2 µM), BIM-1 (5 µM), or TAPI-1 (10 µM). Images were acquired every hour up to 48 hpi, and the mean green fluorescent object area per image was quantified as a measure of syncytia size using IncuCyte software v10.4.1. All live-cell imaging experiments were performed in three independent experiments with three biological replicates (*n* = 9 per condition).

### Western blot

A549 WT and A549 ADAM17^−/−^ cells were seeded at 2 × 10^5^ cells/well in 24-well plates and grown to confluence. Cells were lysed using M-PER Mammalian Protein Extraction Reagent (Thermo Fisher Scientific, cat. no. 78501), according to the manufacturer’s instructions. The total protein concentration was quantified using the Quick Start Bradford Protein Assay (Bio-Rad). Total protein (25 µg per lane) was resolved using 10% SDS-PAGE under reducing conditions and transferred to a PVDF membrane using a Trans-Blot Turbo Transfer System (Bio-Rad). Membranes were incubated with primary antibodies diluted in a Blotting Accelerator (PR20039, Proteintech), which combines the blocking and primary antibody incubation steps. The following primary antibodies were used: anti-ADAM17 (clone JM10-35, MA5-32572, Invitrogen; 1:1,000), Direct-Blot HRP-conjugated anti-β-actin (clone W16197A, cat. no. 664804, BioLegend; 1:16,000), and polyclonal goat anti-RSV (AB1128, Merck; 1:2,500). The RSV structural proteins G, F, and N were identified based on their expected apparent molecular weights. HRP-conjugated secondary antibodies were applied for 1 h at room temperature; HRP-conjugated recombinant rabbit anti-mouse IgG (H+L) (A27025, Invitrogen; 1:5,000) was used to detect anti-ADAM17, and HRP-conjugated donkey anti-goat IgG (H+L) (A15999, Invitrogen; 1:10,000) was used to detect the anti-RSV antibody. Signals were detected using chemiluminescence and visualized using a ChemiDoc MP imaging system (Bio-Rad).

### Surface ADAM17 quantification

A549 WT and A549 ADAM17^−/−^ cells were seeded at 2 × 10^5^ cells per well in 24-well plates and infected with rRSV-A-0594-eGFP at MOIs of 0.1 and 1, or mock-treated. At 48 hpi, cells were fixed as adherent monolayers with 4% paraformaldehyde in PBS for 15 min at room temperature and washed twice with PBS. Non-specific binding was blocked with 5% BSA in PBS for 1 h at room temperature. The cells were then stained with a PE-conjugated mouse anti-human TACE/ADAM17 ectodomain monoclonal antibody (clone 111633, FAB9301P, R&D Systems) for 2 h at room temperature in the dark. Surface ADAM17 fluorescence (PE channel, Ex/Em 496/578 nm) and viral load (eGFP channel, Ex/Em 488/520 nm) were simultaneously quantified using a Tecan Spark multiplate reader. The linear relationship between ADAM17-PE and RSV-eGFP mean fluorescence intensities was assessed by linear regression for each genotype, with the slope tested against zero to evaluate the dose-dependent surface ADAM17 redistribution in response to RSV infection.

### Dispase-based epithelial sheet fragmentation assay

To assess epithelial monolayer integrity during RSV infection, A549 WT and A549 ADAM17^−/−^ cells were seeded at 2 × 10^5^ cells/well in 24-well plates and grown to 100% confluence to form a solid monolayer prior to infection. Cells were infected with rRSV-A-0594-eGFP at an MOI of 1 or mock-treated. At 24 hpi, DECMA-1 (clone DECMA-1; cat. no. 16-3249-38; Thermo Fisher Scientific; 10 µg/mL) was added to the designated wells as a positive control for junction disruption. At 48 hpi, the cells were incubated with MTT solution (0.5 mg/mL in PBS) for 2 h at 37°C to stain the entire monolayer, followed by a brief PBS wash. Monolayers were then treated with 0.01% trypsin for 2 min at 37°C to loosen matrix adhesions, followed by aspiration and a PBS wash. Dispase II (1 U/mL in PBS) was added at 300 µL/well and incubated at 37°C until complete detachment of the monolayer as a single cohesive sheet was observed. The detached sheets were then subjected to standardized mechanical shear using an electronic multichannel pipette (Eppendorf Xplorer, speed 5, 300 µL, 10 pipetting strokes per well). Fragmentation of the epithelial sheets was qualitatively assessed from brightfield images acquired using a Leica DM8 fluorescence microscope.

### Immunofluorescence and confocal microscopy

To investigate the junctional distribution of E-cadherin and vimentin during RSV infection, A549 WT and A549 ADAM17^−/−^ cells were seeded on 8-well chamber slides (Thermo Fisher Scientific, cat. no. 155409PK) at 2 × 10⁴ cells per well and infected with rRSV-A-0594-eGFP at an MOI of 1 or mock-treated. Cells were fixed at 48 hpi with 4% PFA in PBS for 10 min at room temperature and blocked with 5% BSA in PBS for 1 h. Primary antibodies were applied overnight at 4°C: anti-vimentin (clone V9, Thermo Fisher Scientific, cat. no. 14-9897-82; 1:100) and anti-E-cadherin (clone HECD-1; Thermo Fisher Scientific, cat. no. 13-1700; 1:100). After washing with PBS, the cells were incubated with a goat anti-mouse IgG H+L cross-adsorbed secondary antibody (Invitrogen, cat. no. A-11005; 1:2,500), washed again with PBS, dried overnight, and mounted using ProLong Glass Antifade mounting medium (Invitrogen). Images were acquired using a TCS SP5 confocal microscope (Leica).

### Transepithelial electrical resistance measurement

Epithelial barrier integrity was assessed by continuous impedance-based transepithelial electrical resistance (TEER) measurements using a cellZscope E system (NanoAnalytics GmbH) operated in a humidified incubator at 37°C with 5% CO₂. A549 WT and A549 ADAM17^−/−^ cells were seeded at 6.5 × 10⁵ cells per insert on the apical side of Sarstedt TC inserts for 6-well plates (cat. no. 83.3930.040, PET membrane, pore size 0.4 µm, tissue-culture treated). Once a stable baseline was established, cells were infected with RSV-A-0594 at MOIs of 0.1 and 1, or mock-infected. The TEER was recorded automatically every 2 h continuously up to 60 hpi and expressed in Ω · cm². Absolute TEER values for A549 monolayers were lower than those typically reported for primary bronchial epithelial cultures, consistent with the comparatively leaky intercellular junctions characteristic of this cell line. Therefore, TEER changes in this study should be interpreted as relative measures of barrier integrity between genotypes and conditions, rather than as absolute measures of epithelial tightness.

### FITC-dextran paracellular permeability assay

To quantify paracellular permeability during RSV infection, A549 WT and A549 ADAM17^−/−^ cells were seeded at 2.5 × 10⁴ cells per insert on Sarstedt TC inserts for 24-well plates (cat. no. 83.3932.040, PET membrane, pore size 0.4 µm, tissue-culture treated) and allowed to adhere for 24 h. Cells were infected with RSV-A-0594 at MOIs of 0.1 and 1 for 2 h. Where indicated, recombinant human amphiregulin (40 ng/mL) was added to the apical chamber immediately following infection. At 0, 24, and 48 hpi, FITC-dextran (4 kDa; 1 mg/mL stock, diluted 1:25 in FluoroBrite DMEM) was applied to the apical chamber (100 µL per insert), and fresh FluoroBrite DMEM (600 µL) was added to the basolateral compartment. After 90 min of incubation at 37°C, 5 × 100 µL of basolateral medium was transferred to a black 96-well plate with optical bottom (Nunc), and fluorescence was measured using a Tecan Spark multiplate reader.

### ELISA-based ectodomain shedding quantification

To quantify ectodomain shedding, conditioned supernatants were collected from A549 WT and A549 ADAM17^−/−^ cells at 0, 24, and 48 hpi from mock-infected cells and cells infected with MOIs of 0.1 and 1. The supernatants were cleared by centrifugation at 300 × *g* for 5 min at 4°C and stored at −20°C until analysis. Shed ectodomains were quantified according to the manufacturer’s instructions using the following kits: Human EGF ELISA Kit (KE00138, Proteintech), Human EGFR ELISA Kit (KE00294, Proteintech), Human Amphiregulin ELISA Kit (RAB0019-1KT, Merck), Human TGF-α ELISA Kit (RAB0459-1KT, Merck), Human MUC5AC ELISA Kit (EEL062, Thermo Scientific), Human E-Cadherin (CDH1) ELISA Kit (EEL016, Thermo Fisher), Human Syndecan-1 ELISA Kit (EHSDC1, Thermo Fisher), Human Syndecan-4 ELISA Kit (EH442RB, Thermo Fisher), Human JAM-A/CD321/F11R ELISA Kit (EH283RB, Thermo Fisher), Human ICAM-1 (Soluble) ELISA Kit (BMS201, Thermo Fisher), Human TLR-2 ELISA Kit (ELH-TLR2-1-RB, RayBiotech), Human IL-6 sR ELISA Kit (ELH-IL6sR-1-RB, RayBiotech), Human IL-1 sRII ELISA Kit (ELH-IL1R2-1-RB, RayBiotech), and Human sTNFRI ELISA Kit (ELH-TNFR1-1-RB, RayBiotech). TNF-α was quantified using the Lumit TNF-α Immunoassay (W6050, Promega). All assays were performed with six biological replicates per condition, and fluorescence or luminescence was measured using a Tecan Spark plate reader.

### Cytotoxicity assessment by lactate dehydrogenase release assay

Lactate dehydrogenase (LDH) release was measured in the cell culture supernatants of A549 WT and A549 ADAM17^−/−^ cells infected with RSV-A-0594-eGFP at MOIs of 0.1 and 1 at 24 and 48 hpi, alongside mock-infected controls, using the Cytotoxicity LDH Assay Kit (MedChemExpress, cat. no. HY-K1090) according to the manufacturer’s instructions. Supernatants were collected in parallel with samples designated for ELISA-based ectodomain shedding analysis to allow direct cross-referencing of the cytotoxicity levels and shedding signals within the same experimental replicates. Results are expressed relative to the WT mock control.

### Transcriptomic profiling by RNA sequencing

To characterize the transcriptional consequences of ADAM17 deficiency during RSV infection, total RNA was extracted from A549 WT and A549 ADAM17^−/−^ cells at 24 hpi (MOI 1) and from mock-infected controls using the NEB Monarch Total RNA Miniprep Kit. Messenger RNA was enriched from total RNA using poly(T) oligo-attached magnetic beads, fragmented, and converted to cDNA by first-strand synthesis using random hexamer primers, followed by second-strand synthesis. Libraries were prepared by end repair, A-tailing, adapter ligation, size selection, amplification, and purification, and sequenced on an Illumina platform (paired-end).

Raw sequencing reads were filtered to remove adapter-contaminated reads, reads containing more than 10% ambiguous nucleotides (*N* > 10%), and low-quality reads in which more than 50% of bases had a Phred quality score of 5. Clean reads were aligned exclusively to the human reference genome (hg38) using HISAT2 (v2.2.1) with default parameters; the RSV genome was not included in the reference genome, and RSV-derived reads were therefore not quantified. All differential expression analyses were restricted to host gene expression. Gene-level read counts were quantified using featureCounts (v2.0.6). Differential gene expression analysis between A549 ADAM17^−/−^ and A549 WT cells under uninfected mock conditions and following RSV infection (24 hpi, MOI 1) was performed using edgeR (v4.0.16) with empirical Bayes dispersion estimation, TMM normalization, and Benjamini-Hochberg (BH) correction for multiple testing. Differentially expressed genes were defined by a dual threshold of adjusted *P*-value ≤ 0.05 and |log2 fold change| ≥ 1 (Table S2). Functional enrichment analysis of differentially expressed genes was performed using clusterProfiler (v4.8.1) for Gene Ontology (GO) and Reactome pathway enrichment (*P*adj ≤ 0.05).

### Statistical analysis

Statistical analyses were performed using GraphPad Prism v10.4.1. Statistical tests are listed in the figure legends, and a *P*-value of <0.05 was considered statistically significant for all analyses.

## RESULTS

### ADAM17 is broadly expressed in various lung cell types and involved in processes related to ectodomain shedding and membrane remodeling

**Fig 1:**
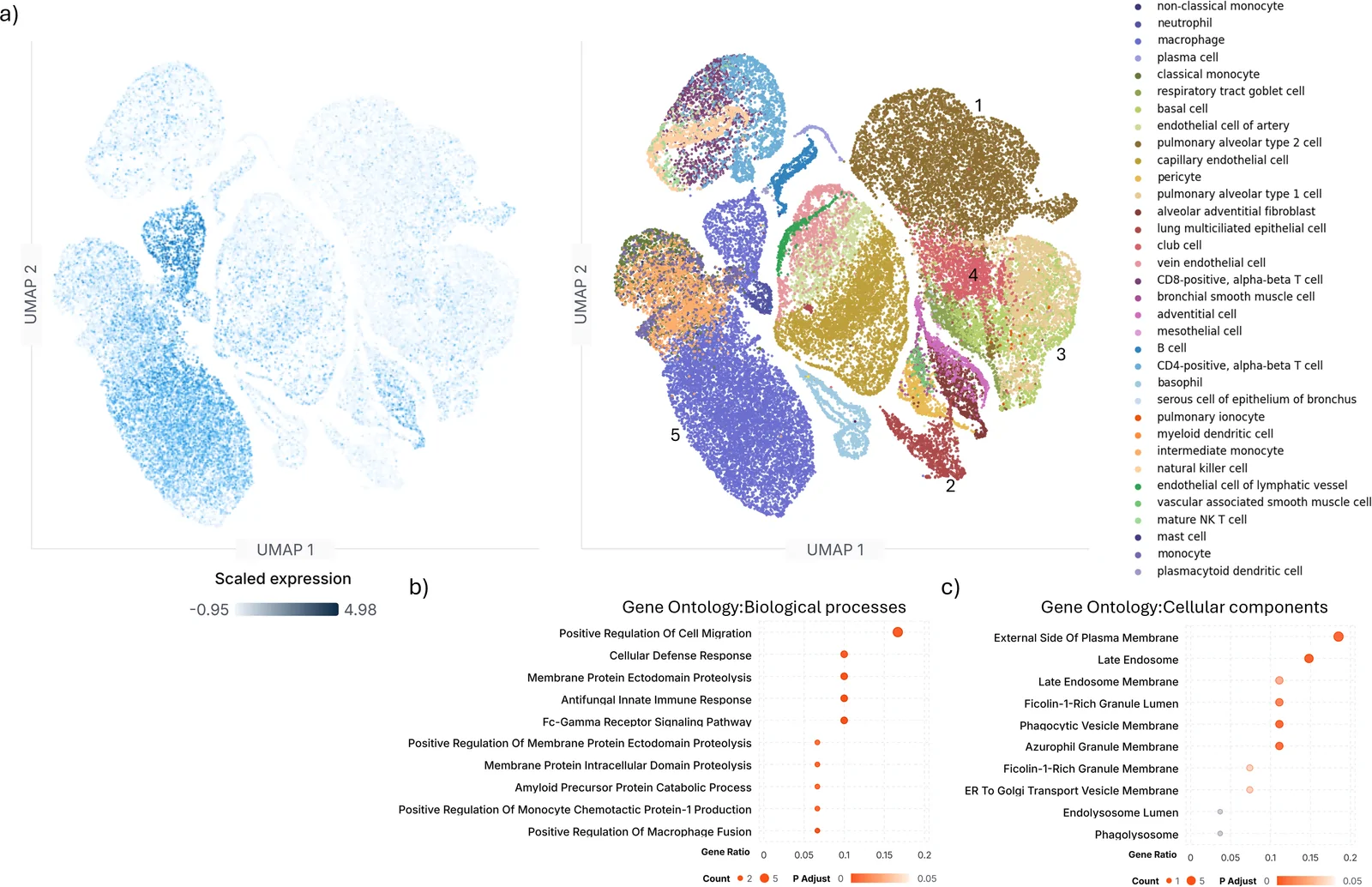
ADAM17 is broadly expressed in RSV target cell populations of the human lung and is linked to ectodomain shedding processes. (**a**) UMAP projection of ADAM17 expression across annotated human lung cell clusters, generated from aggregated single-cell transcriptomic data (Mavatar Discovery platform, Human Lung v1.0 network). Numbered populations indicate the following: 1, Type II alveolar epithelial cells (AT2); 2, ciliated cells; 3, basal cells; 4, club cells; and 5, macrophages. (**b**) Gene Ontology biological process enrichment of the ADAM17 co-expression network (DINA framework), ranked by adjusted *P*-value; enrichment significance calculated using default Gene Ontology enrichment statistical testing. (**c**) Gene Ontology cellular component enrichment of the same network, using the same default statistical approach.

To establish the biological relevance of ADAM17 in pulmonary tissue, we analyzed its expression in various human lung cell types using the Mavatar single-cell transcriptomics platform. The UMAP projection revealed broad ADAM17 expression in both epithelial and immune cells (Fig. 1a). Within the epithelial compartment, its expression was detected in pulmonary alveolar type 2 cells, lung multiciliated epithelial cells, basal cells, club cells, macrophages, and respiratory tract goblet cells. These populations represent the primary target cells of RSV infections in the lower respiratory tract. While ADAM17 is expressed across relevant RSV target cell types, ADAM17 sheddase activity is heavily governed post-translationally by prodomain processing, phosphorylation, and iRhom chaperone trafficking. Gene ontology analysis of biological processes identified positive regulation of membrane protein ectodomain proteolysis and cellular defense response as the most significantly enriched terms, together with positive regulation of monocyte chemotactic protein-1 production and macrophage fusion (Fig. 1b). Cellular component analysis localized ADAM17 to the external side of the plasma membrane and late endosomal compartments, consistent with its function as a surface-resident sheddase that cycles through endosomal recycling pathways (Fig. 1c). Together, these data indicate that ADAM17 is expressed in airway epithelial cell types targeted by RSV and is functionally linked to the ectodomain shedding machinery.

### Generation and characterization of an ADAM17-deficient A549 cell line

**Fig 2:**
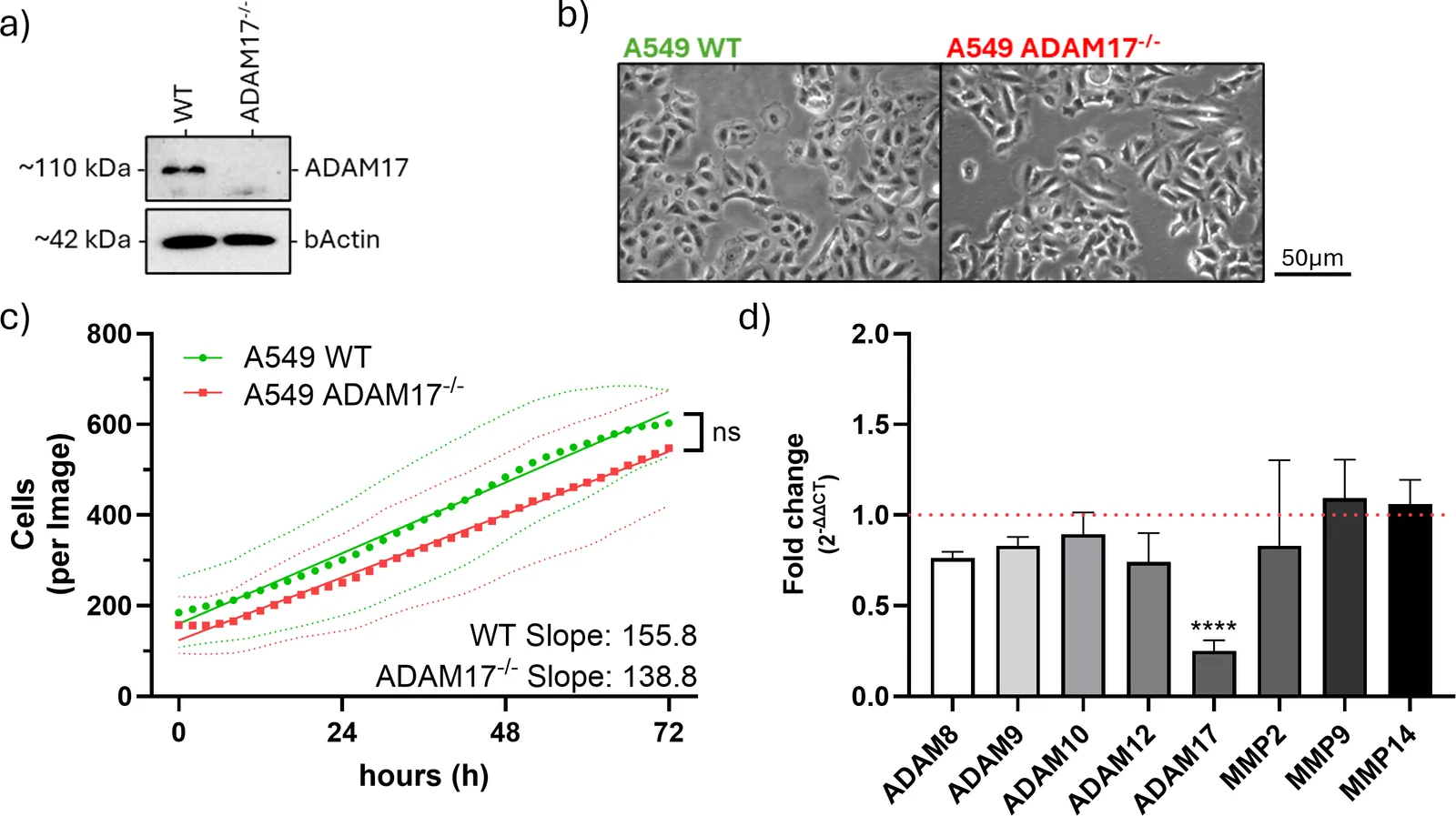
CRISPR-Cas9-mediated ADAM17 knockout in A549 cells does not alter morphology, proliferation, or related sheddase expression. (**a**) Western blot of ADAM17 (110 kDa) and β-actin (loading control) in A549 WT and ADAM17⁻/⁻ cells. (**b**) Representative brightfield images of A549 WT and ADAM17⁻/⁻ monolayers. (**c**) Proliferation kinetics over 72 h quantified by IncuCyte label-free cell counting; slopes compared using simple linear regression with an F-test for equality of slopes (*n* = 9). (**d**) RT-qPCR quantification of ADAM17 and related sheddases (ADAM8, ADAM9, ADAM10, ADAM12, MMP2, MMP9, and MMP14) in ADAM17⁻/⁻ relative to WT cells (2^⁻ΔΔCt^ method, normalized to GAPDH/PGK1/PPIA geometric mean), analyzed by one-way ANOVA. Error bars represent SD (*n* = 6).****P < 0.0001.

To investigate the role of ADAM17 in RSV infection, we generated an ADAM17-deficient A549 cell line (A549 ADAM17⁻) using CRISPR-Cas9 genome editing. Two guide RNAs targeting exon 9 of the ADAM17 locus were used for clonal selection. The resulting clone carried a composite homozygous deletion of exon 9 in all three alleles, as confirmed by Sanger sequencing. Western blot analysis confirmed the complete loss of ADAM17 protein (MW 110 kDa) synthesis, with β-actin as a loading control (Fig. 2a). Brightfield microscopy revealed no morphological differences between A549 WT and A549 ADAM17^−/−^ cells (Fig. 2b). Cell proliferation kinetics over 72 h were comparable between the two lines (slopes 155.8 vs 138.8 cells per image per hour, respectively; *P* > 0.05; Fig. 2c). To assess whether loss of ADAM17 expression induced compensatory upregulation of related proteases, we quantified the mRNA expression of ADAM8, ADAM9, ADAM10, ADAM12, MMP2, MMP9, and MMP14 by RT-qPCR. None of the tested proteases were significantly upregulated in A549 ADAM17⁻ cells. ADAM17 transcript levels were reduced (2^⁻ΔΔCt^ = 0.25; *P* < 0.0001), confirming effective knockout without compensatory sheddase induction (Fig. 2d).

### RSV activates ADAM17 in a MOI-dependent manner via PKC and ERK signaling

**Fig 3:**
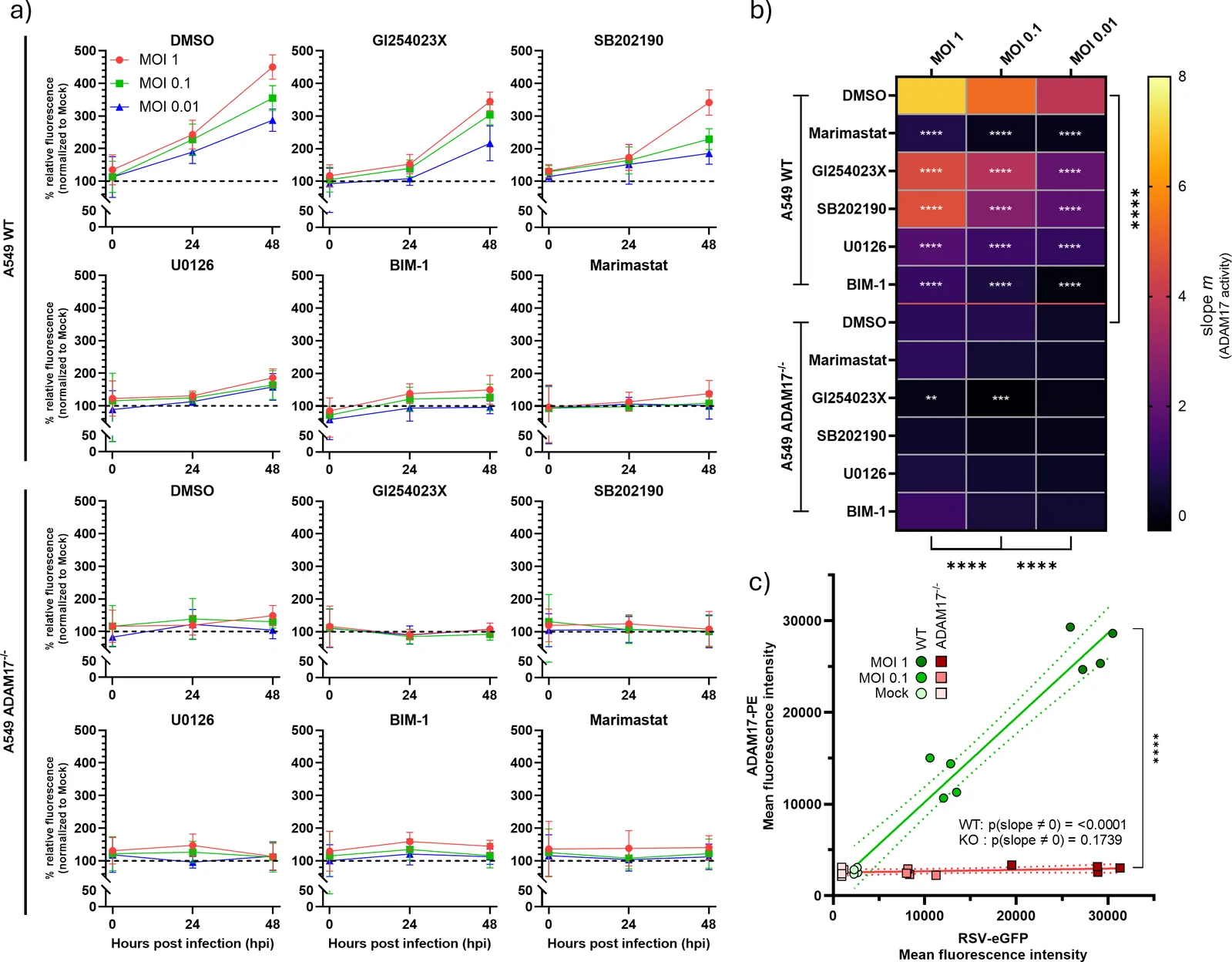
RSV infection triggers MOI-dependent ADAM17 sheddase activity and surface redistribution through a PKC/ERK-dependent pathway. (**a**) FRET-based ADAM17 sheddase activity assay in A549 WT and ADAM17^−/−^ cells infected with RSV-A-0594 (blue, MOI 0.01; green, MOI 0.1; and red, MOI 1) and treated with vehicle (DMSO), Marimastat, BIM-1, U0126, GI254023X, or SB202190; fluorescence measured at 0, 24, and 48 h post-substrate addition (Ex/Em 485/535 nm), normalized to mock. (**b**) Heatmap of linear regression slopes for time-dependent ADAM17 activity accumulation per condition and genotype; slopes were derived by linear regression of the time-course curves in panel a and compared using two-way ANOVA (*n* = 9). (**c**) Surface ADAM17 (PE) versus RSV-eGFP mean fluorescence intensity by simple linear regression in WT and ADAM17^−/−^ cells at 48 hpi (MOI 0.1, 1). Error bars represent SD (*n* = 4). **P < 0.01, ***P < 0.001, and ****P < 0.0001.

To determine whether RSV infection induces ADAM17 sheddase activity and to define the upstream signaling pathway, we performed a fluorogenic ADAM17 activity assay. Infection with RSV-A-0594 at MOIs of 0.01, 0.1, and 1 induced a robust, MOI-dependent increase in ADAM17 activity in A549 WT cells at 48 hpi, reaching up to 400% of the mock-normalized fluorescence at MOI 1 under vehicle control (DMSO) conditions (Fig. 3a). The broad-spectrum metalloproteinase inhibitor Marimastat and the PKC inhibitor BIM-1 both abolished ADAM17 activity at all MOIs tested, while the MEK/ERK inhibitor U0126 produced strong but incomplete inhibition. The ADAM10-selective inhibitor GI254023X and p38 MAPK inhibitor SB202190 produced only modest inhibition. No ADAM17 activity was detected in A549 ADAM17^−/−^ cells at any treatment or MOI, confirming assay specificity (Fig. 3a). Heatmap visualization of the linear regression slopes for time-dependent ADAM17 activity accumulation confirmed this pattern: untreated WT cells reached maximal slope values of 7–8, whereas Marimastat and BIM-1 reduced slope values to zero. As expected, no ADAM17 activity was induced in ADAM17^−/−^ cells (Fig. 3b).

To assess whether RSV infection drives MOI-dependent surface redistribution of ADAM17, WT and ADAM17^−/−^ cells were infected with RSV-A-0594-GFP at MOIs of 0.1 and 1, fixed as adherent monolayers at 48 hpi with 4% PFA to allow surface detection of ADAM17 without permeabilizing the cells, stained with a PE-conjugated anti-ADAM17 antibody, and surface ADAM17 and RSV-eGFP fluorescence were quantified simultaneously. In A549 WT cells, ADAM17-PE mean fluorescence intensity showed a significant positive linear relationship with the RSV-eGFP mean fluorescence intensity (*P*[slope ≠ 0] < 0.0001), indicating that surface ADAM17 expression was MOI-dependent (Fig. 3c). No significant correlation was detected in A549 ADAM17^−/−^ cells (*P*[slope ≠ 0] = 0.1739). Regression slopes differed significantly between WT and ADAM17^−/−^ cells (*P* < 0.0001), confirming that RSV infection induced an MOI-dependent increase in plasma membrane-resident ADAM17.

### ADAM17 is dispensable for RSV entry but promotes viral replication and syncytium formation

**Fig 4:**
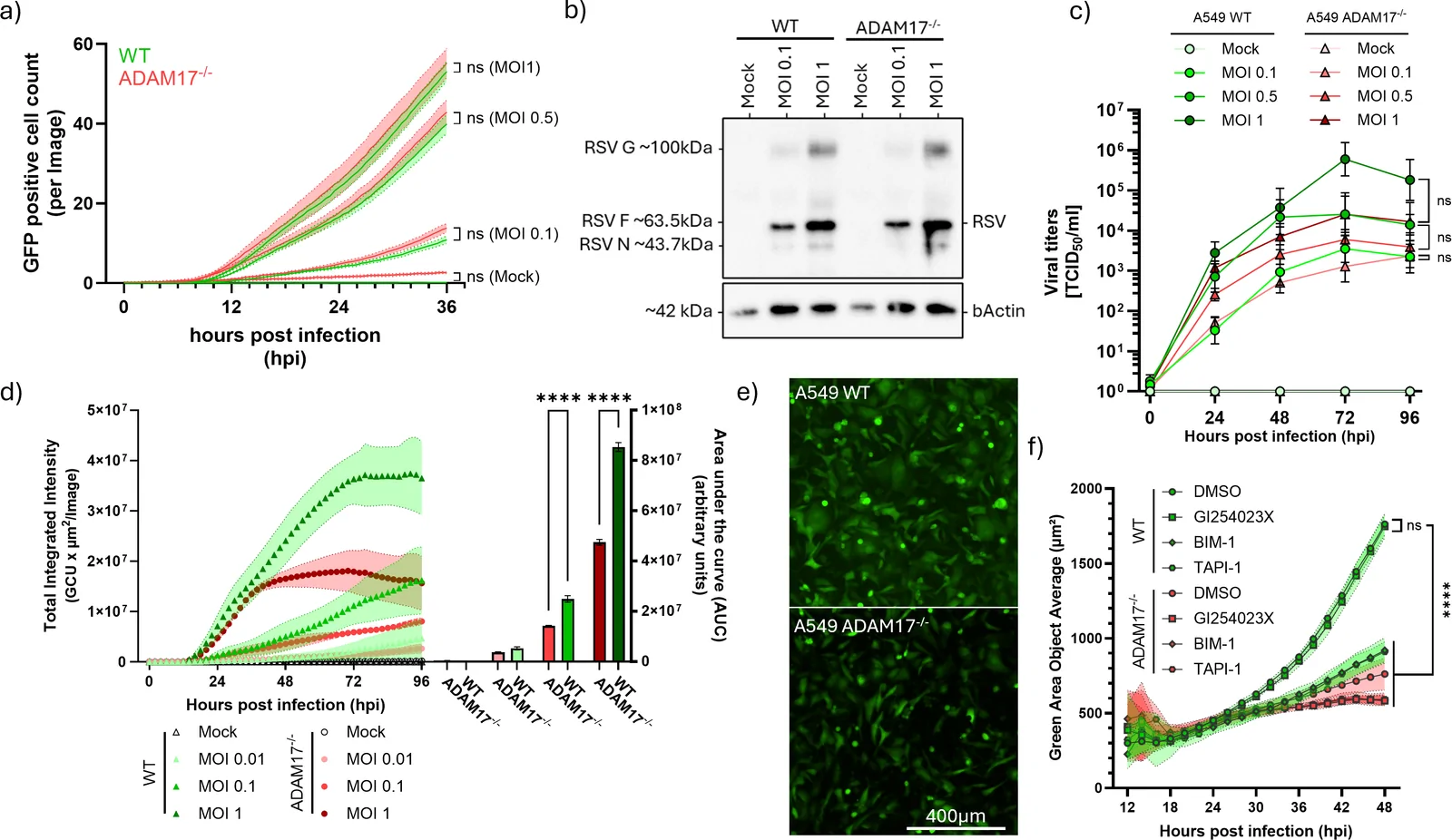
ADAM17 is dispensable for RSV entry but required for efficient post-entry spread and formation of syncytia. (**a**) GFP-positive cell counts by live-cell imaging up to 36 hpi (RSV-A-0594-eGFP, MOIs 0.1, 0.5, and 1) in WT and ADAM17^−/−^ cells (*n* = 6). (**b**) Western blot of RSV structural proteins G (100 kDa), F (63.5 kDa), and N (43.7 kDa) at 36 hpi (MOIs 0.1 and 1); representative of three independent experiments. (**c**) Infectious virus titers in culture supernatants across MOIs and timepoints; virus undetectable in mock (*n* = 9). “ns” indicates that the overall viral replication kinetics across the entire time course do not significantly differ between WT and ADAM17^−/−^ cells (two-way ANOVA with Tukey’s post hoc test; *P* > 0.05); detailed statistical comparisons are provided in Table S1. (**d**) Total integrated GFP intensity time course (0–96 hpi) and area-under-curve quantification of cumulative viral spread (*n* = 6). (**e**) Representative live-cell images at an MOI of 1, 72 hpi showing GFP-positive syncytia; representative of three independent experiments. (**f**) Mean green object area (syncytia size) at MOI 1 with DMSO, GI254023X, BIM-1, or TAPI-1 treatment in WT and ADAM17^−/−^ cells (*n* = 12). All comparisons by two-way ANOVA. Error bars represent SD; all data from three independent experiments. ****P < 0.0001.

To determine whether ADAM17 plays a role in RSV replication and at which step of the viral life cycle, we first compared early infection dynamics in A549 WT and ADAM17^−/−^ cells in a cell entry assay. Upon infection with RSV-A-0594-GFP, GFP-positive cell counts measured by live-cell imaging up to 36 hpi showed no significant differences between A549 WT and ADAM17^−/−^ cell lines at MOIs of 0.1, 0.5, or 1 (*P* > 0.05; Fig. 4a). Western blot analysis of cell lysates at 36 hpi confirmed comparable levels of RSV structural proteins G (100 kDa), F (63.5 kDa), and N (43.7 kDa) in both cell lines at MOIs of 0.1 and 1 (Fig. 4b). Thus, ADAM17 expression did not influence viral entry or early protein synthesis.

After 36 hpi, replication kinetics differed markedly between WT and ADAM17^−/−^ cells. Infectious virus titers in the culture supernatants of WT cells at 72 and 96 hpi were significantly higher than those in ADAM17^−/−^ cells. At MOI 1, titers of 10^5.8^ (72 hpi) and 10^5.3^ (96 hpi) TCID_50_/mL were reached with WT cells compared to 10^4.4^ (72 hpi) and 10^4.2^ (72 hpi) TCID_50_/mL in ADAM17^−/−^ cells, more than a 10-fold difference, respectively. At an MOI of 0.5, titers of 10^4.2^ and 10^3.6^ TCID_50_/mL were observed for WT and ADAM17^−/−^ cells, respectively. At an MOI of 0.1, comparable titers of 10^3.4^ TCID_50_/mL were observed in both cell lines. The infectious virus was undetectable in mock-infected control cell cultures (Fig. 4c). Total integrated viral-driven GFP intensity measurements corroborated these findings. WT cells showed a steep MOI-dependent increase in viral spread from 36 hpi onward, whereas ADAM17^−/−^ cells reached a plateau at substantially lower levels. Area under the curve analysis confirmed significantly greater cumulative replication in WT cells at MOI 1 (8.35 × 10^7^ vs 4.75 × 10^7^ arbitrary units; *P* < 0.0001) and MOI 0.1 than in ADAM17^−/−^ cells (*P* < 0.0001; Fig. 4d). Representative live-cell images at MOI 1 and 72 hpi show large GFP-positive syncytia in WT cells that were absent in ADAM17^−/−^ monolayers (Fig. 4e).

To determine whether ADAM17 sheddase activity specifically supported the formation of syncytia, we quantified the green object area in RSV-A-0594-GFP-infected WT and ADAM17^−/−^ cells treated with pharmacological inhibitors. DMSO- and GI254023X-treated WT A549 cells formed similar large syncytia (mean green object area: 1,762.6 µm² and 1,747.6 µm², respectively), confirming that inhibition of ADAM10 activity did not impair syncytium formation. In contrast, BIM-1, the PKC inhibitor, and TAPI-1, a specific ADAM17 inhibitor, reduced green object area to 920.0 µm² and 910.0 µm², a significant reduction of approximately 48% compared to vehicle control (*P* < 0.0001). A549 ADAM17^−/−^ cells formed substantially smaller green objects regardless of inhibitor treatment (DMSO: 762.3 µm²; GI254023X: 582.9 µm²; TAPI-1: 593.0 µm²; BIM-1: 587.0 µm²). There was no significant difference between the untreated and inhibitor-treated ADAM17^−/−^ cells (*P* > 0.05; Fig. 4f). Specific inhibition of ADAM17 activity in WT cells and absence of ADAM17 activity in untreated ADAM17^−/−^ cells produced the same phenotype. These findings indicate that ADAM17 activity promotes RSV-induced formation of syncytia.

### ADAM17 strengthens epithelial barrier integrity during RSV infection via EGFR ligand shedding

**Fig 5:**
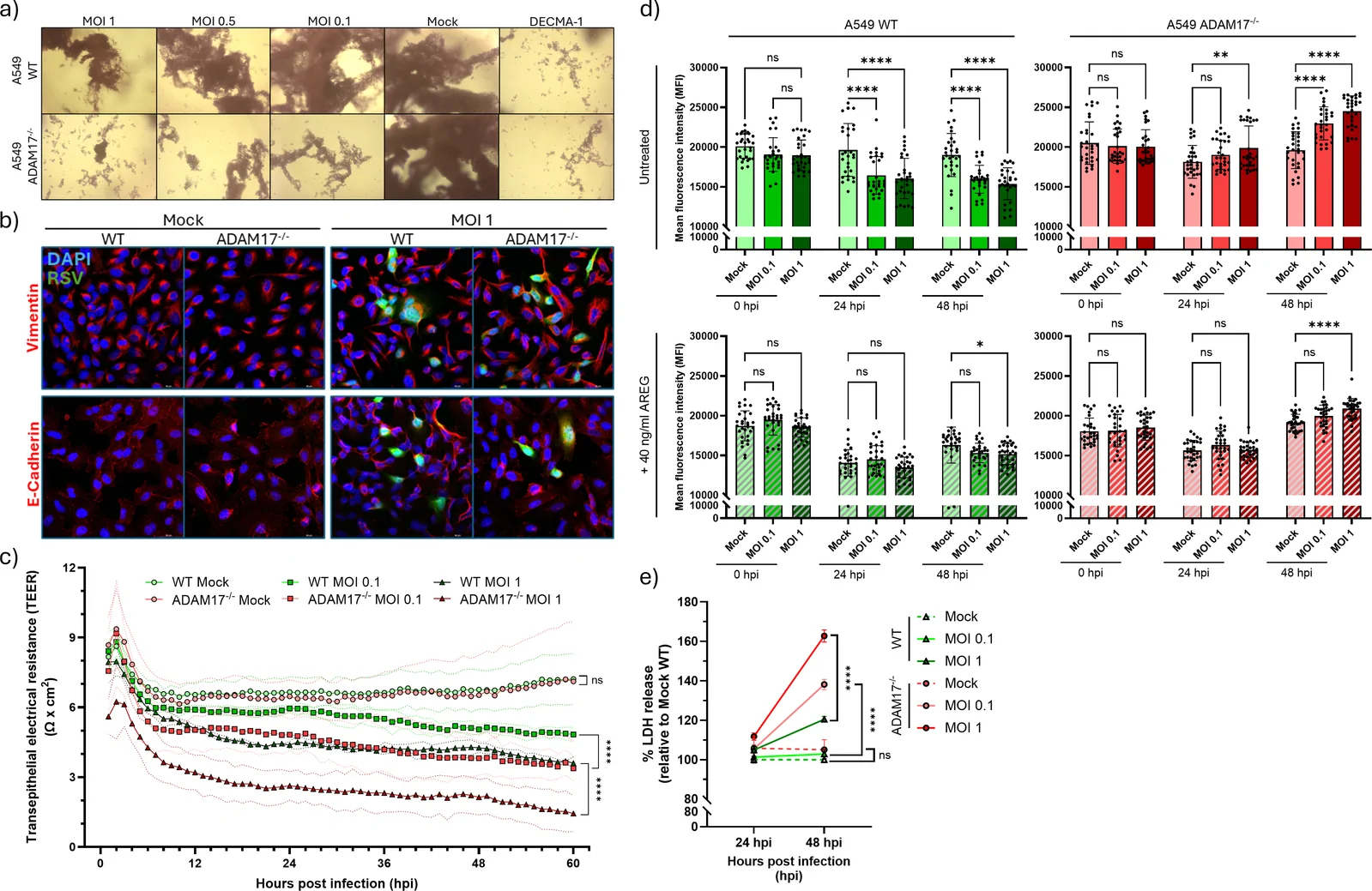
ADAM17 loss compromises intercellular cohesion, junctional E-cadherin polarization, barrier resistance, and cell survival during RSV infection. (**a**) Dispase-based epithelial sheet fragmentation assay; representative brightfield images of mock- and RSV-infected WT and ADAM17^−/−^ monolayers, with DECMA-1 (anti-E-cadherin function-blocking antibody) as positive control for junction disruption (*n* = 6). (**b**) Confocal immunofluorescence of E-cadherin and vimentin in mock- and RSV-infected (MOI 1, 48 hpi) WT and ADAM17^−/−^ cells. (**c**) TEER over 60 hpi (MOIs 0.1 and 1) in WT vs ADAM17^−/−^ cells (*n* = 3). (**d**) Transwell FITC-dextran passage assay (MOIs 0.1 and 1; 0/24/48 hpi) with and without recombinant AREG (40 ng/mL) rescue (*n* = 30). (**e**) LDH release assay (MOIs 0.1 and 1; 24/48 hpi) relative to WT mock (*n* = 9). Statistical comparisons in panels c–e by two-way repeated-measures ANOVA followed by Tukey’s post hoc test. Error bars represent SD. *P < 0.05, **P < 0.01, ***P < 0.001, and ****P < 0.0001.

To understand the mechanism underlying the role of ADAM17 in RSV-induced syncytium formation, we assessed the epithelial barrier integrity in WT and ADAM17^−/−^ cells. First, a dispase-based epithelial sheet fragmentation assay was performed. After mock infection, monolayers of both A549 WT and ADAM17^−/−^ cells formed large cohesive structures that resisted fragmentation (Fig. 5a). Upon RSV infection, monolayers of WT cells partially resisted fragmentation in an MOI-dependent manner. In contrast, monolayers of ADAM17^−/−^ cells progressively disintegrated with increasing MOI, similar to treatment with DECMA-1, an antibody against E-cadherin that blocks its function and was used as a positive control (Fig. 5a). Thus, ADAM17 activity controls maintenance of intercellular cohesion during RSV infection.

To investigate the role of intercellular junctions in the observations described above, we localized E-cadherin and vimentin during RSV infection by confocal microscopy of infected WT and ADAM17^−/−^ cells. Vimentin distribution, included as a mesenchymal marker to exclude epithelial-to-mesenchymal transition as a confounding variable, was comparable between WT and ADAM17^−/−^ cells and did not change upon RSV infection (Fig. 5b). In uninfected cells, E-cadherin localization displayed a diffuse pattern that did not differ between the two cell lines. In WT cells, RSV infection at an MOI of 1 induced a qualitative redistribution of E-cadherin toward cell-cell contact regions, observed in both directly infected and neighboring uninfected bystander cells (Fig. 5b). In ADAM17^−/−^ cells, E-cadherin remained diffusely distributed despite comparable infection rates, and no equivalent redistribution was observed. These findings suggest a paracrine mode of action that is dependent on ADAM17-mediated ligand shedding.

TEER measurements over 60 hpi with RSV-A-0594-GFP confirmed ADAM17-dependent differences in barrier integrity. Both uninfected cell lines had comparable resistance at baseline (WT: 7.2 Ω · cm²; ADAM17^−/−^: 7.1 Ω · cm²). RSV infection reduced TEER in both cell lines in an MOI-dependent manner, but the decline was significantly greater in ADAM17^−/−^ cells. At an MOI of 0.1, the endpoint values were 4.8 Ω · cm² for WT cells versus 3.4 Ω · cm² for ADAM17^−/−^ cells. At an MOI of 1, WT cells still had a TEER value of 3.6 Ω · cm², while that of ADAM17^−/−^ cells declined to 1.4 Ω · cm² (*P* < 0.001 and *P* < 0.0001, respectively; Fig. 5c).

Paracellular permeability was further investigated using a Transwell FITC-dextran passage assay. Upon RSV infection, WT cells displayed a lower FITC-dextran passage than uninfected controls (15,385 vs 19,002 MFI at 48 hpi; *P* < 0.0001), indicating that RSV infection strengthened the barrier. In contrast, the opposite was observed in ADAM17^−/−^ cells, where RSV infection increased dextran passage compared to that in uninfected cells (MFIs of 24,506 and 19,610 for infected and uninfected ADAM17^−/−^ cells, respectively; *P* < 0.0001). Supplementation with 40 ng/mL recombinant AREG (a ligand of EGFR) partially restored the barrier function of RSV-infected ADAM17^−/−^ cells, reducing the passage to 20,910 MFI (*P* < 0.0001; 48 hpi, ADAM17^−/−^ infected MOI 1 vs mock; Fig. 5d). These findings suggest that ADAM17-mediated AREG shedding is important for maintaining the barrier integrity during RSV infection.

RSV-induced cytotoxicity in ADAM17^−/−^ cells was assessed using an LDH release assay. LDH release in mock-infected WT and ADAM17^−/−^ cells was comparable (*P* = 0.6149). Upon infection with RSV-A-0594, LDH release increased in an MOI-dependent manner in both cell lines, but was significantly greater in ADAM17^−/−^ cells at both MOI 0.1 and 1 (*P* < 0.0001 for both; Fig. 5e). At 48 hpi and MOI 1, ADAM17^−/−^ cells released 162% LDH relative to mock WT cells, compared to 121% for WT cells. Compared to uninfected cells, ADAM17^−/−^ cells displayed significant cytotoxicity at both MOI 0.1 and 1, whereas WT cells showed a significant increase in LDH release at MOI 1 only. Thus, ADAM17^−/−^ cells were more sensitive to RSV-induced cell death than WT cells were.

### RSV infection-induced ADAM17 activity results in ectodomain shedding of various substrates

**Fig 6:**
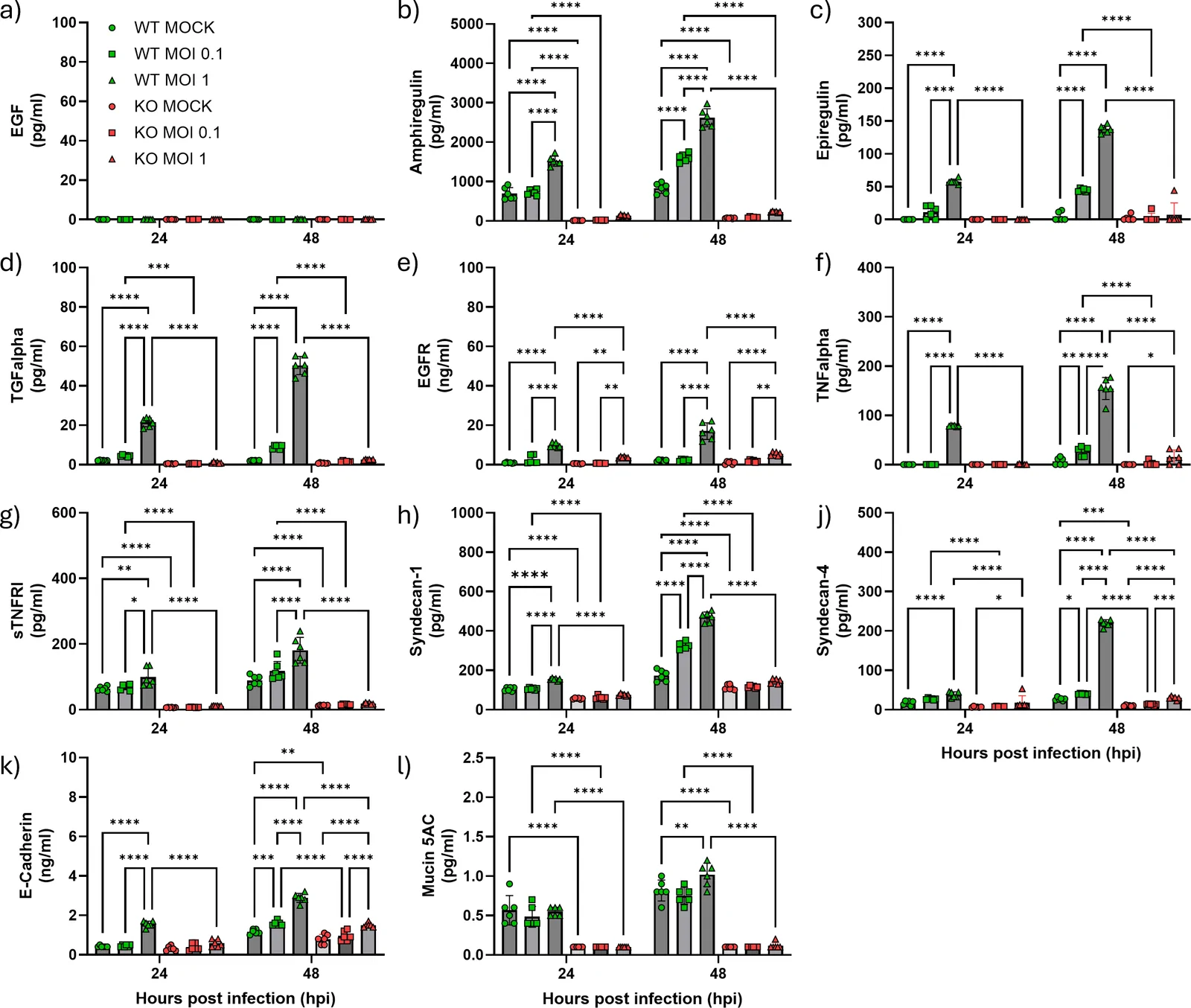
RSV infection drives ADAM17-dependent, MOI- and time-dependent shedding of EGFR ligands (**a–e**), inflammatory mediators (**f, g, l**), viral co-receptors, and adhesion molecules (**h–k**). ELISA/immunoassay quantification of shed ectodomains in culture supernatants of WT and ADAM17^−/−^ cells at 24 and 48 hpi (MOIs 0.1 and 1) and mock. All comparisons by two-way ANOVA. Error bars represent SD. *n* = 6 biological replicates per condition, from three independent experiments. *P < 0.05, **P < 0.01, ***P < 0.001, and ****P < 0.0001.

To quantify ADAM17-mediated shedding during RSV infection, we measured 11 ectodomains released from their substrates by ADAM17 in the culture supernatants of A549 WT and ADAM17^−/−^ cells at 24 and 48 hpi with RSV-A-0594 at MOIs of 0.1 and 1. EGF, included as a negative control for ADAM17 substrate specificity, was undetectable in all the samples tested (Fig. 6a). All measured ADAM17 substrates were readily detected in the supernatants from RSV-infected WT cells, and their release was MOI-dependent. The highest responses were observed at 48 hpi, at an MOI of 1. In contrast, the release of these constituents from ADAM17^−/−^ cells was largely abolished (Fig. 6).

Among the EGFR ligands, AREG was most abundantly released from WT cells, reaching a concentration of 2,617 pg/mL after 48 hpi at an MOI of 1, which was significantly higher than that in ADAM17^−/−^ cells (228 pg/mL; *P* < 0.0001; Fig. 6b). Epiregulin levels reached 138 pg/mL in WT cell cultures after 48 hpi, while it remained virtually undetectable in the culture supernatants of ADAM17^−/−^ cells (*P* < 0.0001; Fig. 6c). TGF-α levels reached up to 50 pg/mL in WT cell cultures but remained very low in the culture supernatants of ADAM17^−/−^ cells (up to 2.4 pg/mL; *P* < 0.0001; Fig. 6d). Concentrations of soluble EGFR ectodomain were elevated in WT cell cultures (17 pg/mL, 48 hpi at an MOI of 1) and were significantly lower after infection of ADAM17^−/−^ cells (Fig. 6e).

TNF-α levels increased to 155 pg/mL at 48 hpi in WT cells, which was significantly higher than that after infection of ADAM17^−/−^ cells (15 pg/mL; *P* < 0.0001; Fig. 6f). The release of soluble TNFRI was also significantly higher in WT cells than in ADAM17^−/−^ cells (181 pg/mL vs 18 pg/mL, respectively; *P* < 0.0001; Fig. 6g).

RSV infection also induced the efficient release of heparan sulfate proteoglycans from WT cells, with concentrations of syndecan-1 and syndecan-4 reaching 470 pg/mL and 220 pg/mL, respectively, at 48 hpi and an MOI of 1. These levels were significantly lower in ADAM17^−/−^ cell culture supernatants (145 pg/mL and 30 pg/mL, respectively; *P* < 0.0001; Fig. 6h through j).

Soluble E-cadherin was shed from WT cells and, to a lesser extent, from ADAM17^−/−^ cells (up to 2.9 ng/mL and 1.5 ng/mL at 48 hpi/MOI 1, respectively; *P* < 0.001; Fig. 6k). MUC5AC was detected in WT cell culture supernatants, including from mock-infected cells, and increased slightly upon RSV infection. In the culture supernatants of ADAM17^−/−^ cells, MUC5AC levels did not exceed the detection limit even after infection with RSV (*P* < 0.05; Fig. 6l).

Collectively, these data show that RSV infection induced MOI-dependent ADAM17-mediated release of ectodomains belonging to the families of EGFR ligands, inflammatory mediators, viral co-receptors, and epithelial adhesion molecules.

### Transcriptional responses to RSV infection in WT and ADAM17^−/−^ cells

**Fig 7:**
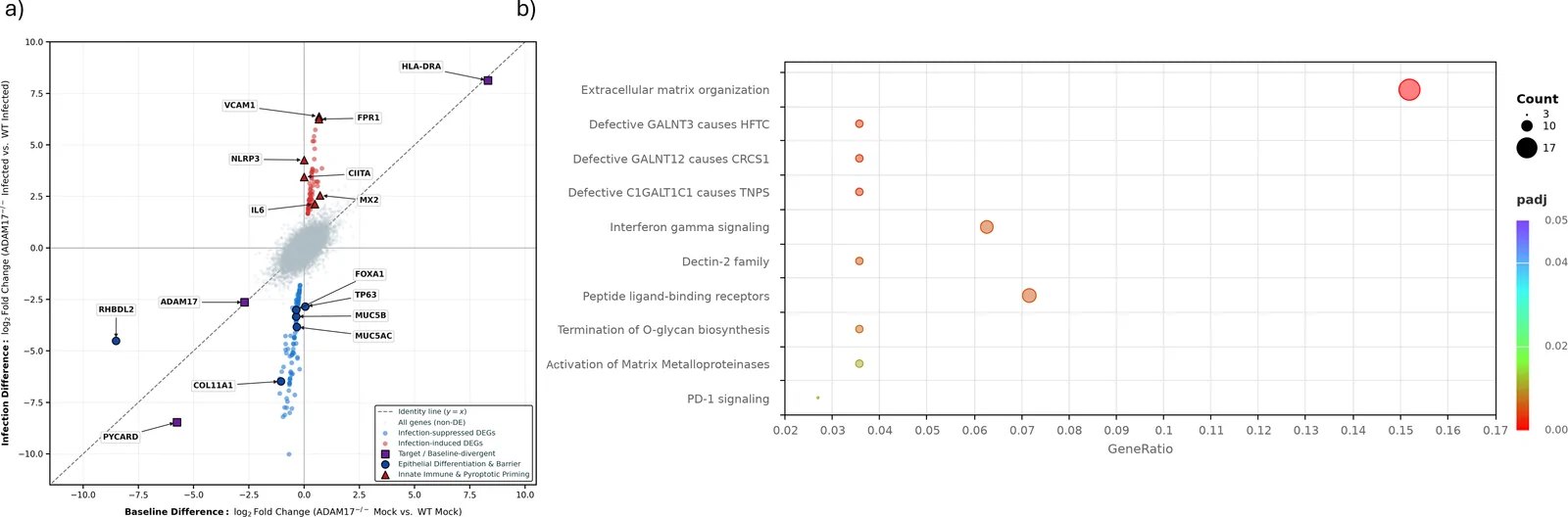
ADAM17 deficiency shifts the RSV-induced transcriptional program from epithelial differentiation toward innate inflammatory signaling. (**a**) Volcano plot of differentially expressed genes (RNA-seq, edgeR, TMM normalization, and BH-corrected) comparing RSV-infected ADAM17^−/−^ vs. WT A549 cells at 24 hpi (MOI 1). (**b**) Reactome pathway enrichment (clusterProfiler, *P*adj < 0.05) of differentially expressed genes, ranked by gene ratio and adjusted *P*-value. *n* = 2 biological replicates per condition (WT/ADAM17^−/−^, mock/infected).

To identify cellular processes linked to ADAM17 expression during RSV infection and to evaluate whether observed differences were infection-induced or reflected baseline clonal variation, we performed RNA sequencing on A549 WT and A549 ADAM17^−/−^ cells under both uninfected mock conditions and following infection with RSV-A-0594 at an MOI of 1 for 24 h. Differential expression analysis using edgeR (FDR ≤ 0.05, |log2FC| ≥ 1; Table S2) identified 194 differentially expressed genes between infected genotypes, of which 120 were more highly expressed in WT-infected cells and 74 in ADAM17^−/−^-infected cells. Plotting baseline fold changes against infection-induced fold changes revealed that while target knockout (ADAM17) and a small subset of baseline-divergent genes (PYCARD, HLA-DRA, and RHBDL2) aligned along the identity diagonal, the primary divergence in epithelial and inflammatory programs occurred specifically upon viral infection (Fig. 7a). A subset of genes was annotated based on their known roles in RSV infection, innate immune signaling, epithelial barrier maintenance, or ADAM17-related biology.

Genes that were expressed to a higher degree in RSV-infected WT cells were enriched for epithelial identity and barrier integrity. Notably, core mucin genes (MUC5AC and MUC5B) and the basal progenitor regulator TP63 were not differentially expressed at baseline (*P*adj > 0.05; Table S2), but were specifically downregulated in ADAM17^−/−^ cells following RSV infection (Fig. 7a). RHBDL2, a rhomboid serine protease involved in EGFR ligand shedding, was among the most strongly downregulated genes in ADAM17^−/−^ cells. The expression of FOXA1 was markedly reduced in ADAM17^−/−^ cells upon infection, consistent with the loss of the AREG-EGFR-ERK feedforward loop that sustains goblet and basal epithelial differentiation. As expected, ADAM17 transcript levels were reduced. COL11A1 and PYCARD were among the most strongly suppressed genes in ADAM17^−/−^ cells. PYCARD encodes the inflammasome adaptor protein ASC, which bridges NLRP3 to caspase-1, and showed a log2FC of −8.46 (Fig. 7a). Genes that were expressed to a higher extent in ADAM17^−/−^-infected cells included NLRP3 (log₂FC = +4.27) and IL-6, indicating a shift toward a pro-inflammatory state in the absence of ADAM17. Both genes showed minimal baseline expression in uninfected controls and were induced by RSV infection to a great extent (Fig. 7A; Table S2). CIITA and HLA-DRA levels were also elevated, consistent with the upregulation of IFN-γ-responsive antigen presentation genes (30). MX2, an interferon-stimulated antiviral effector primarily induced by type I interferons (31), was also upregulated. FPR1, a pattern recognition GPCR that drives neutrophil and monocyte chemotaxis toward danger-associated signals (32), and VCAM1, an NF-κB- and IL-6-regulated leukocyte adhesion molecule (33), showed the highest differential expression in RSV-infected ADAM17^−/−^ cells (Fig. 7a).

Reactome pathway enrichment analysis identified extracellular matrix organization as the most significantly enriched pathway (GeneRatio ≈ 0.155; *n* = 17 genes), driven by the downregulated gene cluster in ADAM17^−/−^ cells, and consistent with disrupted epithelial matrix remodeling. IFN-γ signaling and peptide ligand-binding receptors were enriched within the upregulated inflammatory gene set. The activation of matrix metalloproteinases and PD-1 signaling was also enriched (Fig. 7b).

## DISCUSSION

In the present study, we identified ADAM17 as an RSV-induced host factor important for maintaining epithelial barrier integrity and promoting syncytial formation. RSV infection induced ADAM17 activity via an ERK-PKC-dependent signaling pathway. The sheddase activity of ADAM17 strengthened barrier integrity through paracrine EGFR ligand signaling and facilitated post-entry viral spread and formation of syncytia.

Consistent with the established paradigm that ADAM17 transcript abundance does not directly correlate with enzymatic activity, ADAM17 activity is controlled primarily at the post-translational level through its regulatory co-factors iRhom1 and iRhom2, which regulate trafficking to the plasma membrane and substrate accessibility, with iRhom1 predominating in epithelial cells and iRhom2 predominating in immune cells (24, 34–36). PKC and ERK phosphorylate both the cytoplasmic tail of ADAM17 and iRhom co-factors, increasing membrane levels and enzymatic activity (24, 37). We found that ADAM17 activity induced by RSV infection was completely abolished by PKC inhibition and strongly suppressed by ERK inhibition, which agrees with previous findings on the role of ERK and PKC in controlling ADAM17 activity (37–39). ADAM10 inhibition had no effect on ADAM17 activity. Thus, during RSV infection, the ERK-PKC axis controls the induction of ADAM17, which is the dominant sheddase responsible for shedding EGFR ligands including AREG and TGF-α (12). In RSV-infected WT cells, cell surface expression of ADAM17 correlated positively with RSV-eGFP signal in an MOI-dependent manner, indicating that higher viral loads are associated with an increased presence of ADAM17 at the cell surface. Because we studied epithelial cells only, the interplay between epithelial iRhom1-ADAM17 and immune cells iRhom2-ADAM17 during RSV infection remains to be elucidated (24, 34–36). Innate activation through TLR3 or RIG-I in macrophages recruited to infected tissues could drive a parallel iRhom2-dependent sheddase response, with distinct substrate preferences and inflammatory outcomes (40).

Although we showed that PKC drives ADAM17 activation during RSV infection, the identity of the upstream trigger remains unknown. PKC-α is activated by RSV in human bronchial epithelial cells and colocalizes with viral particles within minutes of infection, suggesting receptor-proximal interactions (41). RSV also activates NF-κB through PKC and reactive oxygen species, independent of phospholipase C (42–44). PKCδ regulates RSV-induced HIF-1α translation through the PI3K/Akt/mTOR axis, confirming that RSV engagement with PKC produces multiple downstream biological outcomes (45). Whether the initial trigger of PKC activation is F protein-mediated membrane fusion (46), G protein receptor engagement (42, 47), or pattern recognition downstream of RIG-I or TLR3 (40) remains unknown. Notably, other respiratory viruses can also activate ADAM17. The SARS-CoV-2 spike protein drives ACE2 and TNF shedding by ADAM17 (16, 48), influenza A virus infection upregulates ADAM17 in small airway epithelial cells (49), and PRRSV infection increases ADAM17 activity in alveolar macrophages (50). For none of these viruses has the upstream trigger of ADAM17 activation in airway epithelium been identified.

We investigated whether ADAM17 affects RSV infection at the entry stage or at a later stage of the viral replication cycle. Because ADAM17 is expressed on the epithelial cell surface and sheds proteins and heparan sulfate proteoglycans that serve as viral attachment co-receptors (25), we hypothesized that it might affect the attachment and entry of viral particles. A comparison of the early infection dynamics of WT and ADAM17-deficient cells showed that the number of infected cells and the expression of RSV structural proteins G, F, and N were similar up to 36 hpi. These findings indicate that ADAM17 does not play a major role during the early stages of the viral replication cycle. At later time points, RSV replicated and spread significantly better in WT cells than in ADAM17-deficient cells up to 96 hpi. Large syncytia formed readily in WT cells but not in ADAM17-deficient cells, and pharmacological inhibition of ADAM17 activity in WT cells resulted in patterns similar to those observed in ADAM17-deficient cells, whereas ADAM10 inhibition did not. We hypothesize that the role of ADAM17 in later stages of the replication cycle involves ADAM17-mediated remodeling of the cell surface. ADAM17 sheds soluble E-cadherin in an RSV-infection- and MOI-dependent manner, which could reduce junctional adhesive tension and lower the energy barrier for F-protein-driven membrane fusion between neighboring cells (46, 51). Furthermore, ADAM17 sheds the primary heparan sulfates syndecan-1 and syndecan-4, which serve as (co-)receptors for RSV. Their shedding during active RSV infection can segregate cell-free virions and remove steric obstacles for cell-to-cell fusion, favoring cell-to-cell spread (46, 51). Together, E-cadherin and syndecan processing may increase local junctional plasticity and reduce the resistance to syncytium formation (52). Because the extent of ADAM17 expression at the cell surface correlates with the viral load, this may create a positive feedback loop in which the cell surface becomes progressively more permissive to further fusion and lateral spread (51). ADAM17 sheddase activity, which promotes syncytium formation, also had a major effect on epithelial barrier integrity. Although RSV infection can disrupt epithelial barrier integrity by reducing tight junction protein expression (8), WT cells retained barrier integrity better than ADAM17-deficient cells, as shown by the dispase fragmentation assay, TEER measurements, and FITC-dextran passage assay. RSV infection caused redistribution of E-cadherin to cell-cell contact regions in both RSV-infected and uninfected bystander WT A549 cells, indicating that paracrine signaling took place. The addition of exogenous AREG partially restored the barrier integrity in RSV-infected ADAM17-deficient cells, as shown by the FITC-dextran passage assay. Thus, ADAM17-deficient cells, which are impaired in shedding EGFR-stimulating ligands, benefit from the addition of exogenous AREG. Complete restoration of barrier integrity in ADAM17-deficient cells may require additional EGFR ligands, such as TGF-α and epiregulin.

Previously, it was shown that EGFR signaling plays a role in the stress response during chronic airway disease (23). Duox1-derived ROS, neutrophil elastase, and luminal ATP drive PKC-dependent ADAM17 activation, resulting in the release of TGF-α and AREG, which transactivate EGFR to induce MUC5AC production via ERK (21, 53). Similarly, after RSV infection, we observed an MOI-dependent increase in MUC5AC levels in the culture supernatants of WT cells, but not in those of ADAM17-deficient cells. Although MUC5AC is not a direct product of ADAM17 proteolytic activity, it is likely induced indirectly through ADAM17-mediated AREG release and the subsequent AREG-EGFR-ERK signaling axis. Chronic hyperactivation of this axis underlies mucus hypersecretion and remodeling in chronic obstructive pulmonary disease and cystic fibrosis (23, 54). RSV infection also activates this pathway via the induction of ADAM17 activity, resulting in shedding of various EGFR ligands, which drives MUC5AC production and strengthens epithelial barrier integrity.

Together, these findings establish ADAM17 as a host factor supporting RSV infection. ADAM17-deficient cells displayed reduced formation of syncytia and production of infectious virus in cell culture supernatants, especially at later time points post-infection. The reduced viral replication in these cells correlated with increased cell death upon RSV infection, even at an MOI of 0.1. In contrast, ADAM17-mediated EGFR signaling in WT cells resulted in better cell survival and sustained viral replication. These findings are in agreement with those of previous studies showing that RSV infection can activate EGFR in the airway epithelium, delay apoptosis, and sustain viral production (55).

Transcriptomic profiling at baseline prior to RSV infection and in RSV-infected WT and ADAM17-deficient cells was consistent with the functional phenotypes observed and extended these findings to the gene regulatory level. Comparative analysis of uninfected mock controls revealed that baseline expression of core epithelial differentiation markers (TP63, MUC5AC, and MUC5B) and inflammatory alarmins (NLRP3, IL6, VCAM1, and FPR1) was comparable between the two cell lines (Table S2), demonstrating that their subsequent divergence is specifically driven by viral infection rather than pre-existing clonal traits. Genes expressed to a higher degree in RSV-infected WT cells, including TP63 (56), FOXA1 (57), MUC5AC (58), MUC5B (58), and RHBDL2 (59), define the EGFR-ERK-dependent network that sustains goblet and basal epithelial identity (60, 61). Their coordinated downregulation in ADAM17-deficient infected cells is consistent with the absence of AREG-driven EGFR-ERK signaling from these differentiation pathways. The upregulated cluster of genes in ADAM17-deficient cells included NLRP3 (42), IL6, FPR1 (32), VCAM1 (33), HLA-DRA (30), CIITA (30), and MX2 (31), reflecting the amplification of inflammasome activity, leukocyte recruitment signaling, antigen presentation, and interferon-stimulated gene expression (42, 62–64). While our transcriptomic screen utilized duplicate biological libraries per condition, the primary regulated pathways, including the suppression of the AREG-EGFR-MUC5AC axis and elevated inflammatory mediator release, were independently confirmed across multiple independent biological replicates by RT-qPCR (Fig. 2d), multi-analyte ELISA (Fig. 6), and barrier integrity assays (Fig. 5). NLRP3 upregulation is of particular interest in this context, as we have previously shown that the RSV soluble G protein primes NLRP3 via TLR2-MyD88-NF-κB signaling, sensitizing airway epithelial cells to caspase-1-dependent pyroptosis, thereby promoting viral dissemination (42). Elevated NLRP3 expression in ADAM17-deficient cells may therefore reflect a similarly primed, pro-pyroptotic state that contributes to the increased cytotoxicity observed by LDH release. Notably, this upregulation occurred alongside downregulation of PYCARD (ASC), the adaptor required for canonical NLRP3 inflammasome assembly, raising the question of whether the inflammatory cell death in ADAM17-deficient cells proceeds through a canonical, ASC-dependent route, as in our previous model, or through an ASC-independent, non-canonical mechanism.

ADAM17 has been proposed as a driver of pathological inflammation in SARS-CoV-2 infection (65) and chronic lung disease (19, 66), and thus a candidate therapeutic target. However, ADAM17 plays a more ambivalent role in RSV infection. Loss of ADAM17 signaling reduced syncytium formation and viral replication but also impaired epithelial barrier integrity, increased cytotoxicity, and dysregulated innate inflammatory gene expression. Pharmacological ADAM17 inhibition during acute RSV infection, therefore, seems unattractive, since any antiviral benefit would come at the cost of host-protective epithelial function. Whether individuals with naturally reduced ADAM17 activity are more susceptible to severe RSV disease remains unclear. ADAM17 promoter polymorphisms have been linked to altered inflammatory responses in sepsis (67) and to COVID-19 severity (65); however, comparable RSV data are lacking. Our findings suggest that reduced epithelial ADAM17 activity may weaken barrier integrity and shift the epithelial response toward a more inflammatory state, reflected by increased NLRP3- and IL6-associated gene expression in ADAM17-deficient cells; conversely, high ADAM17 activity may favor more efficient viral spread.

In this study, we investigated the role of ADAM17 during RSV infection using a contemporary clinical RSV subgroup A strain of the ON-1 lineage. While sequence divergence between RSV A and RSV B is concentrated in the attachment glycoprotein (G) and may influence attachment kinetics or receptor usage, ADAM17 is an invariant host factor. Its activation and subsequent EGFR ligand shedding represent an intrinsic epithelial response to viral infection and cell-to-cell fusion. Furthermore, both subgroups share functionally homologous F proteins that drive syncytium formation. Therefore, differences in G protein sequence are unlikely to alter the host signaling cascades controlling ADAM17 activity, suggesting that our findings are broadly applicable to subgroup B infections as well. Future studies with contemporary RSV-B isolates will help clarify whether strain-specific replication kinetics influence the extent of ADAM17 induction.

Collectively, we showed that RSV infection in A549 cells induced ADAM17 activity. This metalloproteinase acts as a sheddase on the cell surface and is responsible for the release of various ectodomains belonging to families of EGFR ligands, inflammatory mediators, viral co-receptors, and epithelial adhesion molecules. RSV-induced ADAM17 activity was associated with sustained epithelial barrier integrity, cell survival, efficient syncytium formation, and virus replication, all of which were impaired in ADAM17-deficient cells. Thus, we have identified ADAM17 as an important host factor that supports RSV replication in human airway epithelial cells. Our findings warrant further investigation to confirm the role of ADAM17 in primary bronchial and small airway epithelial cells and in the pathogenesis of RSV infections *in vivo*.

## Data Availability

All data sets are available upon reasonable request. Raw RNA-seq data generated in this study are publicly available in the NCBI Sequence Read Archive under BioProject accession number PRJNA1517699.

## SUPPLEMENTAL MATERIAL

Supplemental material jvi.01150-26-s0001.pdf Fig. S1 and Table S1.

Table S2 jvi.01150-26-s0002.xlsx Complete differential expression dataset of host genes in wild-type and ADAM17^−/−^ A549 cells under baseline mock and RSV-infected condition.

ASM does not own the copyrights to Supplemental Material that may be linked to, or accessed through, an article. The authors have granted ASM a non-exclusive, world-wide license to publish the Supplemental Material files. Please contact the corresponding author directly for reuse.
