# Supplementary material for: Respiratory syncytial virus-induced ADAM17 activity controls maintenance of epithelial barrier integrity and promotes syncytium formation
Source: J Virol. 2026 Sep 28;100(10):e01150-26. doi: 10.1128/jvi.01150-26 (PMC13712789; doi:10.1128/jvi.01150-26)
Supplement: Supplementary file 1 [file jvi.01150-26-s0001.pdf]

Supplemental Material:
Respiratory syncytial virus-induced ADAM17 activity controls maintenance of epithelial barrier integrity and promotes syncytium formation

Supplemental Figure 1:

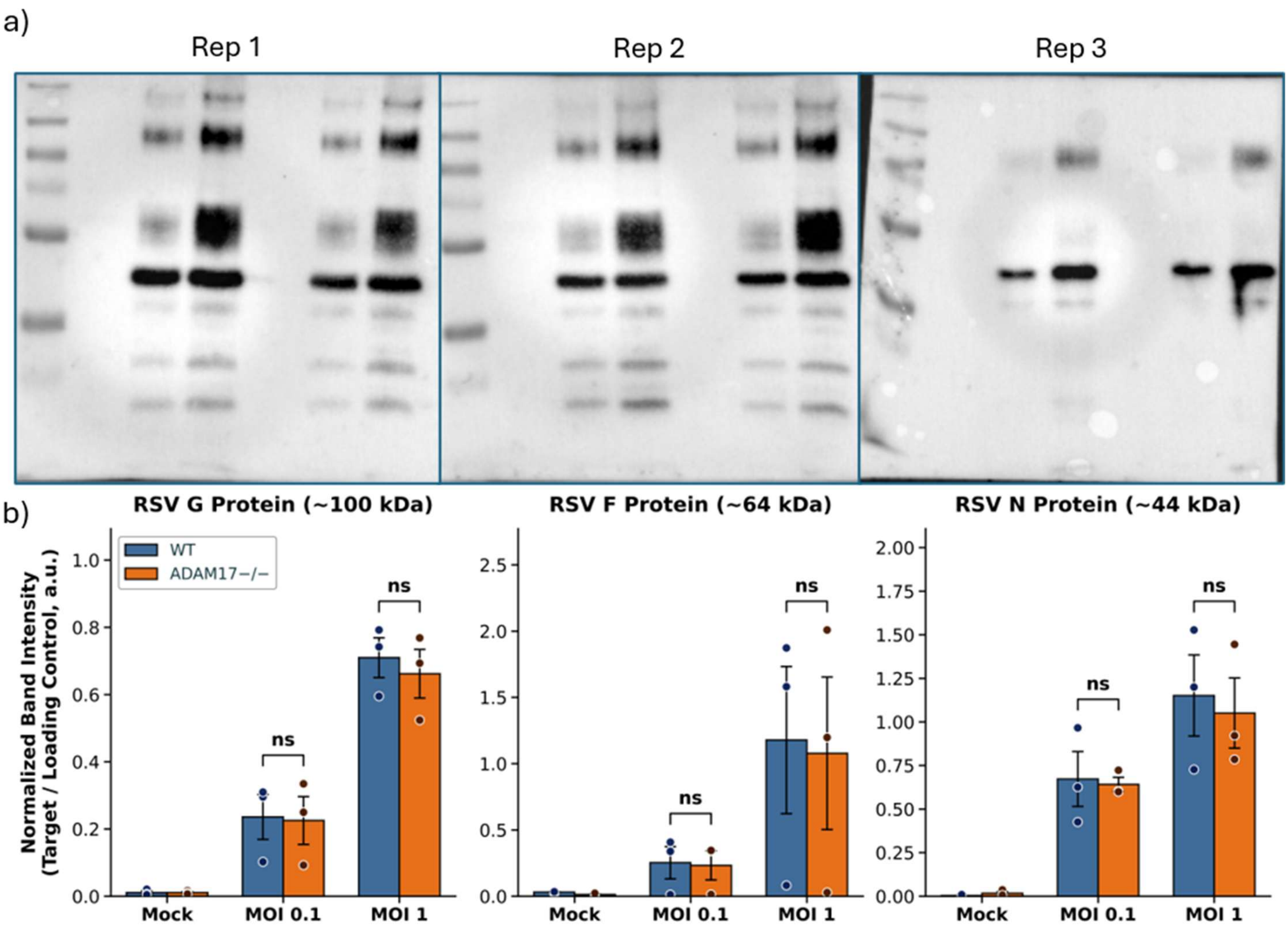

Figure S1. Western blot analysis and densitometric quantification of RSV structural protein levels in wild-type and ADAM17$^{-/-}$ A549 cells. (a) Western blots of whole-cell lysates from wild-type (WT) and ADAM17−/− A549 cells at 36 h post-infection following mock inoculation or infection with RSV at MOIs of 0.1 and 1.0. Membranes were probed against all RSV antigens including G (~100 kDa), F (~64 kDa), and N (~44 kDa). (b) Densitometric quantification showing normalized band intensities (target viral protein adjusted volume / loading control adjusted volume, in arbitrary units [a.u.]) for RSV G (left), F (middle), and N (right) across three independent biological replicates (n = 3). Bars represent the mean ± SEM, with individual biological replicates plotted as discrete data points. Statistical comparisons between WT and ADAM17$^{-/-}$ cells at each MOI were performed using two-way repeated-measures ANOVA with Sidak's multiple-comparisons test; ns, not significant ($p > 0.05$).

Supplemental Table 1:

Condition		Log$_{10}$ TCID$_{50}$/mL A549 WT		Log$_{10}$ TCID$_{50}$/mL A549 ADAM17$^{-/-}$		Two-way ANOVA	
MOI	hpi	Mean	Std. Dev.	Mean	Std. Dev.	p-value	Significance
0.1	0	0.17	0.22	0.07	0.13	0.8154	ns
	24	1.52	0.72	1.70	0.27	0.6402	ns
	48	2.97	0.80	2.70	0.56	0.4956	ns
	72	3.55	0.83	3.11	0.86	0.2709	ns
	96	3.35	1.02	3.37	0.63	0.9624	ns
0.5	0	0.26	0.35	0.00	0.00	0.5133	ns
	24	2.85	0.87	2.41	0.38	0.2628	ns
	48	4.33	1.04	3.41	0.54	0.0200	*
	72	4.41	1.27	3.78	0.57	0.1131	ns
	96	4.15	0.61	3.59	0.82	0.1618	ns
1	0	0.13	0.17	0.13	0.17	>0.999	ns
	24	3.44	0.64	3.07	0.40	0.3506	ns
	48	4.57	1.10	3.85	0.76	0.0692	ns
	72	5.78	1.01	4.41	1.05	0.0006	***
	96	5.26	1.19	4.22	1.09	0.0093	**

Table S1. Infectious viral titers in culture supernatants of A549 wild-type (WT) and ADAM17$^{-/-}$ cells following infection with RSV-A-0594 at MOIs of 0.1, 0.5, and 1 across indicated hours post-infection (hpi). Data represent the mean and standard deviation (SD) from three independent experiments (n = 9 biological replicates per group). Pairwise statistical comparisons between genotypes at each individual timepoint within each MOI were conducted using two-way ANOVA (*, $p < 0.05$; **, $p < 0.01$; ***, $p < 0.001$; ns, $p > 0.05$). Undetectable viral titers in mock-infected controls are omitted.

Supplemental Table 2:

Table S2. Complete differential expression dataset of host genes in wild-type and ADAM17$^{-/-}$ A549 cells under baseline mock and RSV-infected conditions (provided as a separate file: Table_S2.xlsx). Comprehensive listing of all 194 significant differentially expressed genes (DEGs) identified by edgeR (padj < 0.05 and |log2FC| ≥ 1.0; n = 2) comparing ADAM17$^{-/-}$ vs. WT under uninfected mock conditions and following RSV infection (24 hpi, MOI 1). For each gene, the dataset lists the Ensembl gene ID, canonical gene symbol, functional description, normalized mean expression levels (counts per million, CPM), log2 fold changes (log2FC), raw p-values, Benjamini-Hochberg adjusted false discovery rates (padj), and functional biological categories.
